# Localized Enzyme-Assisted Self-Assembly in the Presence of Hyaluronic Acid for Hybrid Supramolecular Hydrogel Coating

**DOI:** 10.3390/polym13111793

**Published:** 2021-05-29

**Authors:** Jennifer Rodon Fores, Alexis Bigo-Simon, Déborah Wagner, Mathilde Payrastre, Camille Damestoy, Lucille Blandin, Fouzia Boulmedais, Julien Kelber, Marc Schmutz, Morgane Rabineau, Miryam Criado-Gonzalez, Pierre Schaaf, Loïc Jierry

**Affiliations:** 1 Institut Charles Sadron (UPR22), Université de Strasbourg, CNRS, 23 rue du Loess, CEDEX 2, BP 84047, 67034 Strasbourg, France; jennifer.rodon-fores@tum.de (J.R.F.); abigosimon@unistra.fr (A.B.-S.); wagner.deborah1@gmail.com (D.W.); mathilde.payrastre@gmail.com (M.P.); camille.damestoy@gmail.com (C.D.); lucille.blandin@gmail.com (L.B.); boulmedais@unistra.fr (F.B.); kelber@unistra.fr (J.K.); marc.schmutz@ics-cnrs.unistra.fr (M.S.); 2 Institut National de la Santé et de la Recherche Médicale, INSERM Unité 1121, CRBS, 1 rue Eugène Boeckel, CEDEX, 67085 Strasbourg, France; morgane.rabineau@inserm.fr; 3 Faculté de Chirurgie Dentaire, Université de Strasbourg, 8 rue Sainte Elisabeth, 67000 Strasbourg, France

**Keywords:** supramolecular hydrogel, enzyme-assisted self-assembly, hyaluronic acid, peptide, coating

## Abstract

Hydrogel coating is highly suitable in biomaterial design. It provides biocompatibility and avoids protein adsorption leading to inflammation and rejection of implants. Moreover, hydrogels can be loaded with biologically active compounds. In this field, hyaluronic acid has been largely studied as an additional component since this polysaccharide is naturally present in extracellular matrix. Strategies to direct hydrogelation processes exclusively from the surface using a fully biocompatible approach are rare. Herein we have applied the concept of localized enzyme-assisted self-assembly to direct supramolecular hydrogels in the presence of HA. Based on electronic and fluorescent confocal microscopy, rheological measurements and cell culture investigations, this work highlights the following aspects: (*i*) the possibility to control the thickness of peptide-based hydrogels at the micrometer scale (18–41 µm) through the proportion of HA (2, 5 or 10 mg/mL); (*ii*) the structure of the self-assembled peptide nanofibrous network is affected by the growing amount of HA which induces the collapse of nanofibers leading to large assembled microstructures underpinning the supramolecular hydrogel matrix; (*iii*) this changing internal architecture induces a decrease of the elastic modulus from 2 to 0.2 kPa when concentration of HA is increasing; (*iv*) concomitantly, the presence of HA in supramolecular hydrogel coatings is suitable for cell viability and adhesion of NIH 3T3 fibroblasts.

## 1. Introduction

Supramolecular hydrogels resulting from the self-assembly of low molecular weight hydrogelators (LMWH) in water [1] give rise to self-assembled nanostructures, mainly nanofibers, following the same mechanisms as those governing classical polymerizations. That is why this self-assembly process is sometimes called supramolecular polymerization [2]. The non-covalent nature of the interactions between LMWH can ensure both a reversibility between the “sol” and the “gel” state, and self-healing features of the resulting material [3]. In addition, the very low amount of hydrogelator used (from 0.01 to 1 wt% in general) leads to a particular class of highly hydrated and thus very soft materials. Peptides-saccharide derivatives have been intensively reported as efficient LMWH for biomedical applications such as wound healing, antibacterial coating, biosensing, 3D cell culture, immunological modulation, drug delivery or tissue engineering [4,5,6,7,8]. Many physical or chemical stimuli were reported to trigger the hydrogelation process. Among them, we can distinguish the use of enzymes since this way involves the chemical transformation of molecular precursors in hydrogelators. These latter are thus produced in situ. In 2004, the group of Bing Xu (Brandeis University, Waltham, MA, USA) introduced the first self-assembly triggered by an enzyme leading to a supramolecular hydrogel [9]. This opened the route to the precise localization of peptide self-assemblies, and a step that was taken in 2009 when Williams and Ulijn groups showed that peptide self-assembly can be initiated from covalently immobilized enzyme on a surface [10]. Our group further developed the localized enzyme-assisted self-assembly (LEASA approach by introducing seed-layers in addition to an enzyme-adsorbed layer to trigger the localized self-assembly growth [11,12], for catalytic and auto-catalytic self-assembled hydrogels [13,14,15] or self-assemblies initiated from nanoparticles surface [16] and in host materials [17,18]. This bottom-up approach is efficient in water and once the surface coated with an adequate enzyme, the localized self-assembled structure growth proceeds in an autonomous way [19].

**Figure 1:**
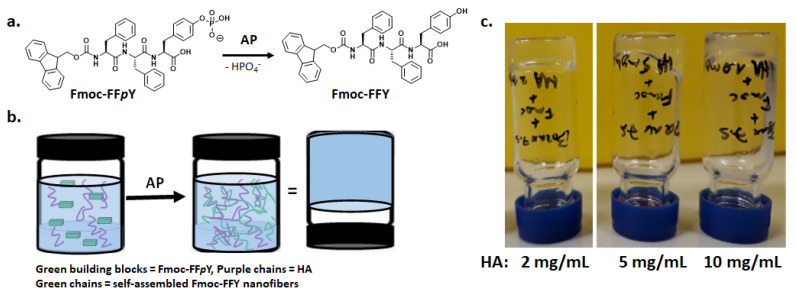
(**a**) Chemical structure of the precursor peptide Fmoc-FF*p*Y transformed in hydrogelator Fmoc-FFY in presence of an enzyme, i.e., AP; (**b**) Scheme of inverted tube test of a Fmoc-FFY hydrogel formed from Fmoc-FF*p*Y precursor and AP in presence of HA; (**c**) Inverted tube tests of self-assembled Fmoc-FFY hydrogels formed in Borax buffer solution (pH 9.5) in presence of various HA concentrations.

Peptide supramolecular hydrogels have exhibited excellent biological properties. Based on the exceptional ability of the FF moiety to self-assemble in various nanostructures [20], the dipeptide Fmoc-FF hydrogel was investigated for 2D and 3D cell cultures (Fmoc = Fluorenylmethyloxycarbonyl; F = phenylalanine) [21,22,23,24]. The analogous morphological features of the nanofibrous self-assembled network of Fmoc-FF with the natural components of the extracellular matrix (ECM) has led to extensive research targeting the design of several peptide hydrogels for specific cell culture lines or biological applications [23,24,25,26]. One interesting aspect of peptide-based hydrogels is the possibility to easily tune both the chemistry and the mechanical features of the resulting material through the co-assembly of different peptide sequences [21,22]. Because the RGD amino acid sequence is a natural endogenous ligand of integrins, a protein receptor involved in the adhesion mechanism of cells, the Fmoc-RGD tripeptide has been largely investigated (R = arginine; G = glycine; D = aspartic acid) [27,28]. Once included in the self-assembled nanostructured of several peptides, Fmoc-RGD is exhibited at the surface of interwoven cylindrical nanofibers and thus promotes adhesion, spreading and proliferation of cells [27]. Longer peptides such as RGDS-Q_11_ and IKVAV-Q_11_ have been also co-assembled leading to β-sheet organization in nanofibers (S = serine; Q = glutamic acid; I = isoleucine; K = lysine; V = valine; A = alanine) [29,30] and showing excellent properties for cell culture support applications [31,32,33,34,35,36,37]. Apart from pure peptide based hydrogels, obtained through different peptide mixtures to modify the properties of the co-assembled structures, hybrid hydrogels can be achieved by combination of peptides and polysaccharides. Among them, hyaluronic acid (HA), a natural negatively charged polysaccharide constituted of repeated disaccharide units, i.e., D-glucuronic acid and N-acetyl-D-glucosamine, attracts great attention in the biomedical field. This polymer is involved in cell signaling, wound reparation, tissue regeneration, morphogenesis and extracellular matrix (ECM) organization in humans. Stupp and coworkers highlighted the interaction of HA with positively charged peptide self-assemblies through the preparation of stable membranes [38,39]. Thin self-supporting bioactive membranes combining positively charged multi-domain self-assembling peptides and HA prepared by varying time and temperature, lead to membranes with finely controlled morphology, which improves the stem cell adhesion and spreading, [40] for tissue regeneration applications. [41] Recently, Nevo and Adler-Abramovich have studied the hybrid supramolecular matrix designed from HA and the dipeptide Fmoc-FF, prepared in a mixture of dimethyl sulfoxide (DMSO) and water [42,43]. The ratio between HA and Fmoc-FF present in the resulting bulk material determined its mechanical features and its biodegradability. However, toxicological properties of DMSO make it an undesirable candidate for the design of many biocompatible hydrogels. For such purpose, herein we use a water-soluble tripeptide, Fmoc-FF*p*Y (Y = tyrosine; *p* = phosphate group), by incorporation of the phosphorylated tyrosine amino-acid to the peptide sequence. Then, this water soluble Fmoc-FF*p*Y precursor is dephosphorylated in presence of alkaline phosphatase (AP) forming the hydrogelator Fmoc-FFY (Figure 1a). We have applied the LEASA concept to direct the hydrogelation process exclusively from the surface of polymer multilayer films, and evaluated the compatibility of this enzyme-assisted self-assembly process with the presence of HA to get hybrid peptide/polymer supramolecular hydrogels with tunable mechanical and biological properties. When adsorbed on a surface through an adequate multilayer film, AP initiates the self-assembly process leading to a micrometer thick hydrogel layer. The influence of HA proportion both on the resulting thickness and on the internal architecture of the peptide self-assembled network was observed by scanning electronic microscopy (SEM and cryo-SEM) and by atomic force microscopy (AFM). The impact of HA on the peptide-based nanofibers organization was studied by transmission electronic microscopy (TEM) and fluorescence confocal microscopy. Since the modification of the internal architecture of hydrogels, the variation of the mechanical properties according to the HA concentration was measured by dynamic oscillatory rheology. Finally, the cell adhesion and cell viability properties of these supramolecular peptide/polymer hydrogels were determined.

## 2. Materials and Methods

### 2.1. Materials

A table indicating the commercial sources of all chemicals used in this work is given in the Supplementary Materials (SM). Materials used: confocal microscope from Zeiss (Oberkochen, Germany), rheometer from Malvern (Malvern, United-Kingdom), the scanning electronic microscope from Hitachi (Tokyo, Japan), the transmission electronic microscope from FEI (Oregon, USA), the atomic force microscope from BioScope Catalyst (Bruker corp., Santa Barbara, CA, USA).

#### 2.1.1. Precursor Peptide Fmoc-FF*p*Y and HA

The precursor peptide Fmoc-FF*p*Y has been prepared through solid support chemistry using a “fmoc” strategy. The synthetic pathway of Fmoc-FF*p*Y is shown in Scheme S1 in the SM. All details about the synthetic procedure and characterization of Fmoc-FF*p*Y (^1^H, ^13^C, ^31^P NMR, HPLC analysis given as Figures S1–S4 respectively in the SM) are given in supporting information, HA characterization (^1^H NMR and size exclusion chromatogram shown as Figures S5 and S6 respectively in the EM) as well.

#### 2.1.2. Buffers and Substrates

All polyelectrolytes and alkaline phosphatase solutions were freshly prepared before being used using a buffer or cell culture medium (RPMI). Borax buffer (25 mM, pH 9.5): 1 g of sodium tetraborate anhydrous was solved in 200 mL of ultrapure water (Milli-Q Plus System, Millipore, Billerica, MA, USA) and pH was adjusted using HCl 0.1 M or NaOH 0.05 M. PBS buffer (pH 7.4): 1 tablet was solved in 200 mL of ultrapure water (Milli-Q Plus System, Millipore, Billerica, MA, USA) and pH was adjusted as described previously. RPMI Cell culture medium was commercially available and used without further steps. Glass slides or silica wafers used for cryo-SEM, confocal, AFM, SAFAS and biological assays, were previously cleaned with an aqueous Hellmanex solution (2% *v/v*) and rinsed extensively with Milli-Q water

### 2.2. Methods

#### 2.2.1. Upside down Test Vials

All tests were performed in glass vials of 1.5 mL capacity. First, four different stock solutions were prepared: a solution of AP at 3 mg/mL (solution 1), a solution of Fmoc-FF*p*Y at 15 mg/mL (solution 2), a solution of HA, three times the desired concentration (solution 3), and finally the borax buffer solution or RPMI (solution 4). All solutions 1, 2 and 3 were prepared in borax buffer. Then, 50 µL of each solution 1, and 2 are mixed together in the glass vial. Then, 50 µL of solution 3 or 4 are added to the previous vial containing solutions 1 and 2 and mixed together with the vortex for 5 s. Then, samples were kept without stirring at room temperature.

#### 2.2.2. Confocal Microscopy

Observations were carried out with a Zeiss LSM 710 microscope using ×10, ×20 and ×40 objectives. To observe the self-assembly, a fluorescent compound, the Thioflavin T (TF), was added to the samples. TF fluorescence was detected between 505 and 530 nm (green emission) after excitation at λ = 488 nm (argon laser). Images were analyzed with Zen black software from Zeiss and “Image J” software. Samples are prepared on a glass surface, by the protocol described in the part 4.3.2, and on solution. Final concentrations in solution are the following: Fmoc-FF*p*Y (5 mg/mL), HA (10 mg/mL), AP (1 mg/mL) and TF (0.15 mg/mL), which is solubilized in the peptide solution. Then, on the glass cover slip 50 µL of each solution (Fmoc-FF*p*Y, TF, AP and HA, or not) are mixed together for the analysis.

#### 2.2.3. Rheological Measurements

Rheological properties were measured on a Kinexus Malvern rheometer using a sand-blasted plate geometry of 1 cm diameter and a gap of 0.5 mm. Firstly, 380 μL of Fmoc-FF*p*Y solution in RPMI were mixed with 20 µL of AP solution (in RPMI as well) within a homemade teflon mold of 1 cm of diameter. The resulting solution was gently shaken for some seconds and left to gelate at room temperature for 24 h. Then, the hydrogel was removed from the mold to perform the measurements. Strain measurements were carried out from 0.01% to 100% at 0.3 Hz and frequency sweeps were performed from 0.01 to 10 Hz at a fixed strain of 0.06%.

#### 2.2.4. Multilayer Film Buildup and Directed Hydrogelation

After deposition of the PEI (1 mg/mL) layer on the chosen substrate by dipping, the multilayer film was performed by alternately exposing the surface to PSS (1 mg/mL in borax buffer) and PAH (1 mg/mL in borax buffer) solutions for 10 min with an intermediate rinsing step with borax buffer for 5 min. Then, AP (1 mg/mL in borax buffer) is adsorbed for 20 min followed by 5 min of rinsing step with borax. Finally, the Fmoc-FF*p*Y solution with or without HA (in borax buffer or in RPMI) was brought in contact with the multilayer polymer film coated substrate for 24 h.

#### 2.2.5. Electronic Microscopy Analyses (TEM, SEM and cryo-SEM)

*SEM and cryo-SEM:* to get cross-sectioned gels, the glass slide (or silica wafer) covered by the supramolecular hydrogel studied, was inserted vertically in the jaws of a homemade vise. The holder with the sample was dipped into the liquid nitrogen slush and then transferred in the chamber of the Quorum PT 3010 machine. High vacuum was applied, and the sample was fractured with an adapted razor blade. After a slight etching at −80 °C the sample was then transferred in the FEG-cryo-SEM (Hitachi SU8010) and observed at 1 kV at −170 °C. *TEM:* hydrogels studied were prepared as described above and then vortexed to get a solution, diluted with borax buffer up to 500 times. 20 µL of a diluted solution was dropped off on a shelf and observed using a TEM Technai G2 machine in negative staining. To make the observations, 5 µL of each solution was deposited onto a freshly glow discharged carbon-covered grid (400 mesh). The solution was left for 2 min and the grid was negatively stained with 5 µL of uranyl acetate (2% in water) for another minute and finally dried using a filter paper. The grids were observed at 200 kV with a Tecnai G2 (FEI) microscope. Images were acquired with a camera Eagle 2 k (FEI) ssCD camera.

#### 2.2.6. Atomic Force Microscopy (AFM)

AFM investigations were carried out with a BioScope Catalyst (Bruker corp., Santa Barbara, CA, USA). Micrographs from different supramolecular hydrogels were recorded in contact mode by using silicon tips mounted on nitride levers. All samples were observed in dry state with triangular cantilevers having a spring constant of 0.4 N/m and a nominal tip radius of 2 nm. Selected AFM images were treated with the nanoscope analysis software (Bruker corp., Santa Barbara, CA, USA). All samples analyzed by AFM were prepared on gold-coated quartz crystal and they were air dried before analysis.

#### 2.2.7. Cell Viability Test

Glass slides of 12 mm of diameter were cleaned up by UV-Ozone for 15 min, and then dropped off at the bottom of a 24-well plate (CORNING brand) for cell culture. Then, the polyelectrolyte multilayer was buildup by filling the 24-wells plate with 1 mL of polyelectrolyte solutions as following. After the deposition of a PEI (1 mg/mL in borax buffer) precursor layer for 10 min, the multilayer film was performed by alternately exposing the surface to PSS (1 mg/mL in borax buffer) and PAH (1 mg/mL in borax buffer) solutions for 10 min with an intermediate rinsing step with borax buffer for 5 min. Then, AP (1 mg/mL in borax buffer) was adsorbed for 20 min followed by 5 min of rinsing step with borax or RPMI medium. Finally, the Fmoc-FFpY solution with or without HA (in borax buffer or RPMI medium) was added and brought in contact for 24 h. Finally, two rinsing steps with the cell culture medium were performed (RPMI + 10% FBS) and then the solution containing the cells were dropped off on the samples. Samples were freshly prepared for the assay and sterilized with UV exposure for 30 min. Then, 5 × 10^4^ NIH 3T3 mouse fibroblasts cells were seeded in each well. The cell culture was done at 37 °C in an incubator for 4 h, in DMEM containing glucose, 10% fetal bovine serum and 1% penicillin-streptomycin. After 4 h, samples were observed using an optical microscope.

## 3. Results

Enzyme-Assisted Self-Assembly (EASA) leads to supramolecular hydrogels differently than with all other methods since the LMWH is generated in situ in water thanks to an enzymatic transformation of a molecular precursor [19]. Many enzymes have proven to be useful on a large variety of precursor compounds. Among them, peptide derivatives are probably the main class of precursors investigated, and thus hydrogelators as well [3]. The precursor is a molecule well soluble in water and the enzymatic action will disturb the balance between its hydrophilicity and its hydrophobicity in favor to this later, driving thus the self-assembly of the hydrogelator [9]. In our study, we have investigated the tripeptide Fmoc-FF*p*Y as precursor and Fmoc-FFY as hydrogelator. This is a model system well studied by our group over the last years [8,11,16,17,18]. When Fmoc-FF*p*Y (5 mg/mL, 6.44 mM) is dissolved in buffer solution at room temperature (Borax buffer 25 mM, pH 9.5, 20 °C), the addition of AP (1 mg/mL, 6.67 µM) induces the self-assembly of Fmoc-FFY in β-sheet organization and then the supramolecular hydrogel is obtained in 20 min. As expected, without the addition of AP, the Fmoc-FF*p*Y solution does not lead to a gel even after two days. To ensure that HA does not prevent the action of AP and/or the gelation process, simple tube test experiments were carried out (Figure 1b). In several vials containing borax buffered solution of Fmoc-FF*p*Y (5 mg/mL), HA (Mw = 406,000 g/mol) was introduced in various proportions to get final 2, 5 and 10 mg/mL concentration of the polysaccharide. After the addition of AP (1 mg/mL), hydrogels were obtained in each vial (Figure 1c) confirming the innocuity of HA toward the hydrogel formation. However, the time required to form the gel is different in each case: ~20, ~40 and ~50 min for the vial corresponding to 2, 5 and 10 mg/mL of HA, respectively. The highest concentration of HA leads to slightly cloudy hydrogels while all others are entirely translucent. This slowdown of the hydrogelation time can be due either to the increase of the viscosity at higher HA concentration or to the fact that HA perturbs the Fmoc-FFY self-assembly and thus the gelation process.

**Figure 2:**
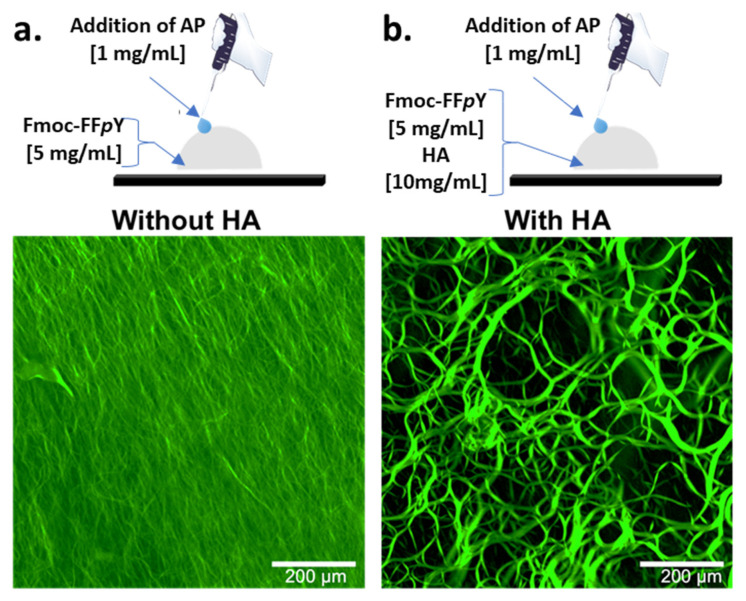
Fluorescence confocal microscopy images taken after the addition of AP (1 mg/mL) in Fmoc-FF*p*Y (5 mg/mL) borax buffered solution (25 mM, pH 9.5) containing Thioflavine T in (**a**) absence or in (**b**) presence of HA (10 mg/mL).

To observe the effect of HA on the self-assembled peptide architecture, we have used fluorescent confocal microscopy which allows observations in hydrogels formed in bulk conditions (vials) and at room temperature. Peptide self-assembly can be revealed by using Thioflavine T (TF), a compound that becomes fluorescent in presence of β-sheet organizations when excited at 450 nm [17]. When Fmoc-FF*p*Y and TF are mixed together (Borax buffer 25 mM, pH 9.5), no fluorescence is measured. The addition of AP solution leads spontaneously to a highly dense-packed tiny microfiber-network emitting a green fluorescence at 482 nm when excited (Figure 2a). When the same experiment is carried out with HA (10 mg/mL) initially present in the precursor Fmoc-FF*p*Y solution, the addition of AP provides a completely different self-assembled microstructure: very long and curvy microfibers are entangled together (Figure 2b). These structures are more than several hundred micrometers long and have diameters ranging from 2 to 15 µm. These experiments support the assessment that the presence of HA considerably impacts the resulting peptide assembly in an EASA process: larger micro-organization occurs in presence of this polysaccharide.

**Figure 3:**
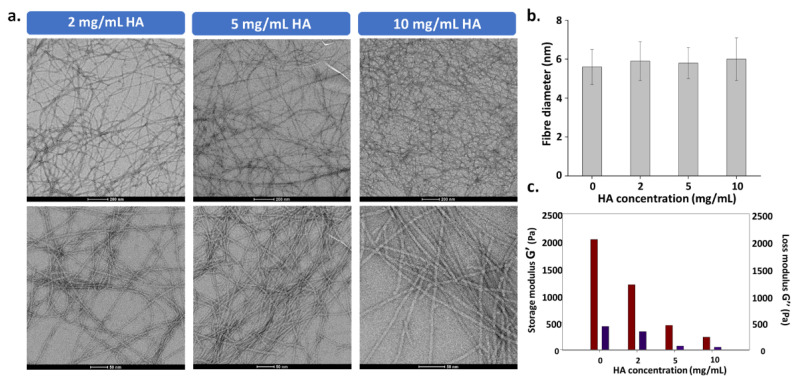
(**a**) The diagram provides the mean diameter of elementary fibers (ca. 30 fibers/image) determined after analysis of several TEM images (ca 10 images); (**b**) TEM images of self-assembled Fmoc-FFY nanofibers formed in presence of different HA concentrations ranging from 2, 5 and 10 mg/mL; (**c**) Elastic modulus (G′) and loss modulus (G″) values measured on hydrogels prepared in presence of 0, 2, 5 and 10 mg/mL of HA.

To get insight into the way HA induces the generation of these microstructures, we examined by TEM the fiber morphology of self-assembled Fmoc-FFY hydrogels in absence or in presence of HA at 2, 5 and 10 mg/mL, in bulk conditions ([AP] = 1 mg/mL, [Fmoc-FF*p*Y] = 5 mg/mL). We focused our attention on the diameter values of the self-assembled single fibers, called elementary fibers (EF). In order to observe them properly, hydrogels were vortexed and diluted with borax buffer before observation. All calculated values result from the mean of thirty measurements at least, randomly selected on several TEM images. Without HA, the self-assembly of Fmoc-FFY leads to elementary nanofibers with 5.8 ± 0.9 nm diameter. When 2, 5 and 10 mg/mL of HA is present with Fmoc-FF*p*Y, the addition of AP leads to EF of 5.9 ± 1.0 nm, 5.8 ± 0.8 nm and 6.0 ± 1.1 nm respectively (Figure 3a,b). Thus, the diameter of EF does not vary when HA is present, even at the highest concentration. Therefore, we can presume that the polysaccharide does not take part in the mechanism of peptide self-assembly initiated by AP but rather interacts with the EF once formed. This seems to indicate that the slow-down of the gel formation observed when the HA concentration is increased is not due to the self-assembly kinetics of Fmoc-FFY, but rather to the interaction of the self-assembled Fmoc-FFY fibers with HA, so that fiber entanglements are less effective.

The change of the peptide self-assembled structure when various concentrations of HA are used can tune the mechanical properties of the resulting hybrid hydrogel as already reported [42,43]. This aspect is particularly important for biological applications since mechanical features of the substrate are directly involved in cell viability, motility and differentiation. Therefore, rheological analyses were carried out using plate-plate geometry. Hydrogels were prepared in home-made Teflon molds providing the right shape and diameter. Anticipating the cell culture study, all hydrogels were prepared in Roswell Park Memorial Institute (RPMI) medium at pH 7.6. The elastic (G′) and loss moduli (G″) were measured at 1 Hz with a shear stress applied of 0.06% (see Figure S7 in SM). The values of both G′ and G″ are given in Figure 3c for each hybrid hydrogel prepared in presence of 0, 2, 5 and 10 mg/mL of HA. When HA is not involved in the hydrogel preparation, the resulting material has a G′ value of roughly 2 kPa. This is an expected order of magnitude for supramolecular hydrogels. By increasing the HA concentration used to get the hydrogel from 2, 5 to 10 mg/mL, the G′ value measured of each hybrid hydrogel decreases to 1.15, 0.47 and 0.20 kPa respectively. G″ values are very low (<0.4 kPa) but follow the same trend. The role of HA proportion on the mechanical features of supramolecular hydrogel agrees with previous and recent contributions based on dipeptide supramolecular hydrogels [42,43].

Circular Dichroism (CD) is a tool to get information related to the chirality at the molecular and supramolecular scale. In particular, it allows for identifying patterns in self-assembled architectures. Thus, when AP is added to a Fmoc-FF*p*Y solution, CD measurements show superhelicoidal structures due to the π-π stacking of the “Fmoc” groups, highlighted by a typical band located at 303 nm [9,44]. In presence of HA (0.5 mg/mL), the whole CD spectra is quasi similar to the one in absence of HA (see Figures S8 and S9 in SM). It appears that the self-assembled Fmoc-FFY organization at the molecular scale is unaffected by HA.

**Figure 4:**
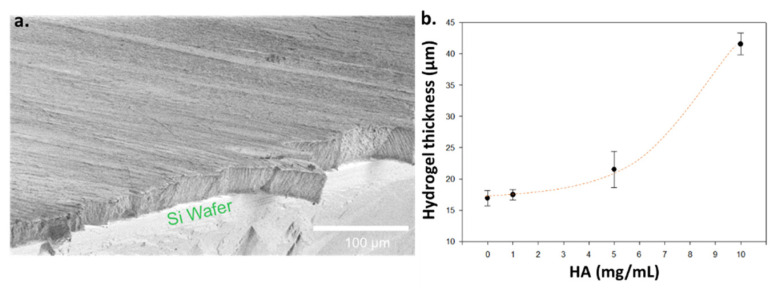

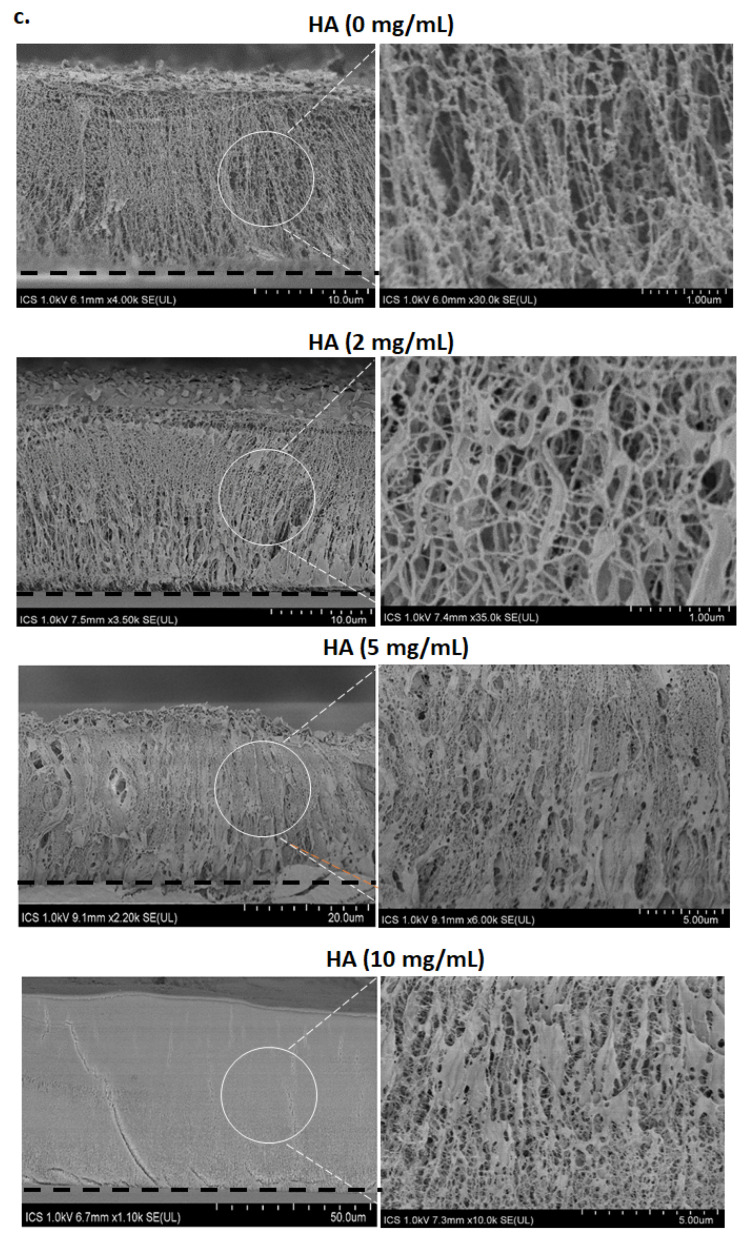
(**a**) Sectional cryo-SEM image of supramolecular hydrogel (dark grey part) formed from silica wafer (light grey part) from Fmoc-FFY self-assembled from PEI/(PSS/PAH)_2_/AP multilayer; (**b**) Hydrogel thickness evolution according to the HA concentration present initially in solution with the precursor peptide Fmoc-FF*p*Y and AP; (**c**) Cryo-SEM images of hydrogels formed from AP-modified multilayer film in presence of various HA concentrations: 0, 2, 5 and 10 mg/mL. Dashed black lines indicate the separation between the glass substrate and the hydrogel.

Localized enzyme-assisted self-assembly is performed when the enzyme is spatially located at a specific area [19,45]. The production of hydrogelators takes place in close vicinity of the enzymes and therefore the hydrogel grows from them. One easy and universal way to get surfaces modified with enzymes is to use multilayer films. This approach is based on the alternation of oppositely charged polyelectrolytes leading to a multilayer film [46]. Enzymes being charged according to the pH value of their environment are able to be adsorbed on top of a film being oppositely charged. AP has an isoelectric point around 5, which means that this enzyme is mainly negatively charged at pH 7.6, i.e., the pH used to build up the thin film. Thus, the following multilayer film has been deposited on silica wafers or glass slides through a dipping process: PEI/(PSS/PAH)_2_ (PEI = poly(ethylene imine); PSS = poly(styrene sulfonate); PAH = poly(allylamine hydrochloride). This multilayer is capped with a polycationic layer due to PAH, allowing the adsorption of AP, and leading to the enzymatically active thin film PEI/(PSS/PAH)_2_/AP. This multilayer architecture ensures to be independent of the substrate’s nature. Then, these polymer multilayer films were dipped in borax buffered solution (pH 9.5) of Fmoc-FF*p*Y (5 mg/mL) containing 0, 2, 5 or 10 mg/mL of HA, during 12 h before analysis by cryo-SEM. To observe both the thickness of each hydrogel formed and its internal architecture, each sample was frozen in liquid ethane, then broken and finally freeze-dried. No metallization has been done. In all studied conditions, cryo-SEM images show a full covering of the substrate by the hydrogel layer (Figure 4a).

**Figure 5:**
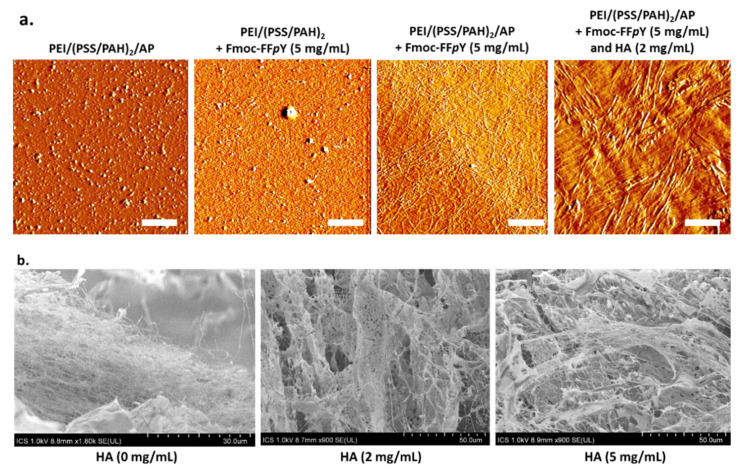
(**a**) AFM images (deflection mode, dry state) of the multilayer film PEI/(PSS/PAH)_2_ in the following conditions (from left to right): after the adsorption of AP on the top of the film, when the multilayer is brought in contact with Fmoc-FF*p*Y solution (5 mg/mL), after the buildup of the peptide self-assembled architecture from a precursor peptide solution [Fmoc-FF*p*Y] = 5 mg/mL in absence or in presence of HA (2 mg/mL). Scale bar = 2 µm; (**b**) SEM images of supramolecular hydrogels formed from PEI/(PSS/PAH)_2_/AP in presence of Fmoc-FFpY (5 mg/mL) and HA at 0, 2 and 5 mg/mL.

Cross-section of the supported self-assembled hydrogels was measured at three different areas at least allowing to represent error bars for each thickness value, highlighting the low roughness of the hydrogel coating all along the substrate through the low standard deviations (Figure 4b). An increase of the thickness is measured from 18 ± 1 µm, 19 ± 0.5 µm, and 22 ± 3 µm to 41 ± 2 µm when the proportion of HA is increased from 0 to 2, 5 and 10 mg/mL respectively. When no HA is present, a fibrillar architecture constituted by nanofibers having a “string of pearls” shape is observed. These fibers are perpendicularly orientated to the substrate, as already reported for others surface-mediated self-assembly of LMWH [11,12,47,48]. When HA is added, the film still displays an internal fibrous organization that remains, at a larger scale, perpendicularly oriented with respect to the substrate. With an increase of the HA concentration, one observes that the fibers are increasingly associated in entities having the shape of veils. These must be certainly constituted both from the Fmoc-FFY self-assembled fibers and HA. As the HA concentration increases, these veils increase in size and fiber network becomes denser. We also performed AFM microscopy on such films. The resulting images were obtained at the dry state. Figure 5a represents typical images obtained in deflection mode. First of all, one observes that PEI/(PSS/PAH)_2_/AP multilayer exhibits aggregates corresponding to clusters of enzymes as already reported by Ulijn and coworkers [10]. In absence of the AP layer over the resulting multilayer film PEI/(PSS/PAH)_2_, no nanofibers are formed once brought in contact with Fmoc-FF*p*Y solution (5 mg/mL), as expected. However, it appears some small globules distributed everywhere on the surface. This could be due to Fmoc-FFpY aggregates as already reported for other Fmoc-peptides hydrogelators. When the enzymatically-active multilayer film PEI/(PSS/PAH)_2_/AP is brought in contact with Fmoc-FF*p*Y solution (5 mg/mL), a dense fibrous network is observed. The presence of HA (2 mg/mL) leads to larger structures that could be formed from the assembly of thin nanofibers in presence of HA. This influence of HA on the internal peptide self-assembled architecture grew up from the surface is confirmed by SEM images taken from surface: kind of veil structures are observed when HA is present (2 or 5 mg/mL) whereas only thinner nanofibers constitute the supramolecular hydrogel in absence of the polysaccharide, as described previously through cryo-SEM studies (Figure 5b).

**Figure 6:**
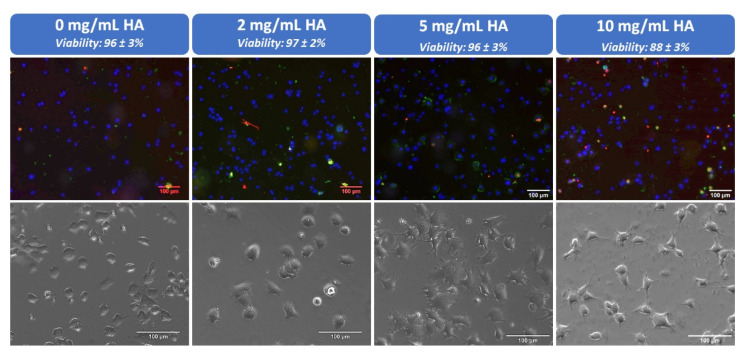
Apoptotic/necrotic/healthy cells detection kit was used to determine the cell viability of NIH 3T3 fibroblasts after 4 h in contact with different hydrogel coatings containing 0, 2, 5 and 10 mg/mL HA. Live cells are stained in blue and death cells in red. Phase contrast microscopy images showing NIH 3T3 fibroblasts cell spreading on the hydrogel coatings.

Finally, biological properties of the developed hybrid peptide/polymer supramolecular hydrogel coatings were assessed. Direct cell adhesion tests were performed by seeding NIH 3T3 fibroblasts on top of the peptide/polymer hydrogel coatings. Fibroblasts (in DMEM + 10% FBS) were brought in contact with the hydrogels for 4 h at 37 °C. Then, a life/dead fluorescent test was performed to obtain the cell viability values for each hydrogel (Figure 6). In comparison with naked glass slides used as control (96.9 ± 1.9% cell viability), cell viability values are excellent for all hydrogels under study with values ≥ 95%, except in the case of the hydrogel containing the highest HA content (10 mg/mL) which is a bit lower (88.4 ± 2.0% cell viability) but suitable for the development of biomaterials. Moreover, cellular spreading was also analyzed through optical image monitoring (Figure 6). When no HA is present, or the concentration is low (2 mg/mL), most of adhered cells shows a round morphology and only few of them have started to spread. When 5 or 10 mg/mL HA are employed, it is possible to distinguish that almost all cells are clearly spread on the hydrogel displaying an elongated shape and philopodes. These results allow us to prove that HA promotes cell adhesion and cell spreading of NIH 3T3 fibroblasts despite the decrease of the mechanical properties of such materials when HA concentration is increased. This achievement is particularly important since previous works showed that NIH 3T3 fibroblasts growth was favored on substrates with high storage modulus.

## 4. Conclusions

The spatial localization of enzymes on polymer multilayer films allows us to direct the peptide self-assembly exclusively where enzymes are located. This LEASA approach is a powerful tool to design micrometer thick supramolecular hydrogel coatings from any kind of substrate using the multilayer film method. Herein, we have used the tripeptide hydrogelator Fmoc-FFY generated in situ from the system AP/Fmoc-FF*p*Y and we have shown that additional polymer components such as HA can be incorporated during the spatially localized hydrogel growth to get hybrid peptide/polymer coatings displaying good cell adhesion and spreading. Thus, HA does not prevent the hydrogelation process and the variation of its proportion allows to modulate the resulting hydrogel thickness. Furthermore, as the HA proportion increased, the storage modulus of the resulting hydrogel decreased, a balance that can be useful to get very soft hydrogels to be employed as essential substrates for nerve cell cultures, for instance. Based on electron and fluorescence microcopy investigations, the incorporation of HA does not change the shape of the peptide-based nanofibers, but it has a strong impact on their ability to collapse together, generating large microstructures. The study of the origin of this interaction between HA and EASA is currently under investigation by our group.

## Data Availability

Data are contained within the article and its Supplementary Materials. Additional data may be provided upon request.

## Supplementary Materials

The following are available online at https://www.mdpi.com/article/10.3390/polym13111793/s1: Scheme S1. Synthetic pathway to prepare Fmoc-FF*p*Y through solid support chemistry; Figure S1. ^1^H NMR spectra of Fmoc-FF*p*Y (400 MHz, CDCl_3_+10%MeOD); Figure S2. ^31^P NMR spectra of Fmoc-FF*p*Y (161.92 MHz, CDCl_3_+10% MeOD); Figure S3. ^13^C NMR spectra of Fmoc-FF*p*Y (100 MHz, DMSO-*d*_6_+10% MeOD) and magnifications; Figure S4. Chromatogram of the purified Fmoc-FF*p*Y; Figure S5. Size exclusion chromatogram of HA; Figure S6. 1H NMR (400 MHz) spectra of HA in D_2_O at 25 °C; Figure S7. Evolution of the elastic modulus (G′) and loss modulus (G″) as a function of the strain (a) or the frequency (b) for Fmoc-FF*p*Y and AP hydrogels in absence or in presence of HA; Figure S8. CD spectra of Fmoc-FF*p*Y and AP in absence (blue line) or in presence (red line) of HA; Figure S9. HT evolution corresponding to the CD spectra of Fmoc-FF*p*Y and AP in absence (blue line) or in presence (red line) of HA.
