# Supplementary material for: Localized Enzyme-Assisted Self-Assembly in the Presence of Hyaluronic Acid for Hybrid Supramolecular Hydrogel Coating
Source: Polymers (Basel). 2021 May 29;13(11):1793. doi: 10.3390/polym13111793 (PMC8198348; doi:10.3390/polym13111793)
Supplement: Supplementary file 1 [file polymers-13-01793-s001.zip › polymers-1241236-supplementary.pdf]

Communication

Localized Enzyme-Assisted Self-Assembly in the Presence of Hyaluronic Acid for Hybrid Supramolecular Hydrogel Coating

Jennifer Rodon Fores [1], Alexis Bigo-Simon [1], Déborah Wagner [1], Mathilde Payrastre [1], Camille Damestoy [1], Lucille Blandin [1], Fouzia Boulmedais [1], Julien Kelber [1], Marc Schmutz [1], Morgane Rabineau [2,3], Miryam Criado-Gonzalez [1,2,3,*], Pierre Schaaf [1,2,3,*] and Loïc Jierry [1,*]

Materials and methods

1. Abbreviations & list of chemicals

AP=Alkaline Phosphatase
CD=Circular Dichroism
SPE tube= Solid Phase Extraction tube
Fmoc =Fluorenylmethoxycarbonyl
NMR= Nuclear Magnetic Resonance
ppm= part per million
HPLC = High performance Liquid Chromatography

All chemicals used in this work are gathered in the following table. They were used as received, without further purifications.

Name, acronym (abbreviation)	Molecular weight($g.mol^{-1}$)	Supplier	CAS Number
**2- Chlorotrityl chloride resin (2-CTC)**	1.6 mmol/g	Iris Biotech (Marktred-witz, Germany)	42074-68-0
**Dichloromethane 99.8% anhydrous (DCM)**	84.93	Carlo Erba (Val-De-Reuil, France)	75-09-2
**N,N-Dimethylformamide 99.8% H_2O = 0.1% anhydrous (DMF)**	73.09	Carlo Erba (Val-De-Reuil, France)	68-12-2
**N-Fmoc-tyrosine(O-phosphate)-OH 98.5% (Fmoc-*p*Y-OH)**	483.41	Bachem (Bubendorf Switzerland)	147762-53-6
**Diisopropylethylamine> 99% (DIEA)**	129.4	Sigma-Aldrich (Saint Quentin Fallavier, France)	7087-68-5
**Methanol RPE ACS 99.9% (MeOH)**	32.04	Carlo Erba (Val-De-Reuil, France)	67-56-1
**Piperidine >99.5%**	85.15	Sigma-Aldrich (Saint Quentin Fallavier, France)	110-89-4
**N-Fmoc-phenylalanine-OH 98% (Fmoc-F-OH)**	387.43	Sigma-Aldrich (Saint Quentin Fallavier, France)	35661-40-6
**2-(1H-Benzotriazol-1-yl)-1,1,3,3-tetramethyluronoium hex-afluorphosphate >98% (HBTU)**	379.25	Alfa Aesar (Kandel, Germany)	94790-37-1
**Hydroxybenzotriazole with no less than 14 wt.% water 98% (HOBt)**	135.12	Sigma-Aldrich (Saint Quentin Fallavier, France)	123333-53-9
**Trifluoroacetic acid 99% (TFA)**	114.02	Alfa Aesar (Kandel, Germany)	76-05-1
**Triisopropylsilane 98%($HSi(iPr)_3$)**	158.36	Sigma-Aldrich (Saint Quentin Fallavier,	6485-79-6

		France)	
**Ethylic ether RPE stabilize BHT (Ether) 99.8% (Et_2O)**	74.12	Carlo Erba (Val-De-Reuil, France)	60-29-7
**Potassium cyanide ACS reagent, ≥96.0% (KCN)**	65.12	Sigma-Aldrich (Saint Quentin Fallavier, France)	151-50-8
**Pyridine extra dry (Py)**	79.10	Prolabo (Sion, Switzerland)	110-86-1
**Ninhydrin 99%**	178.14	Alfa Aesar (Kandel, Germany)	485-47-2
**Butanol ACS reagent, ≥99.4% (BuOH)**	74.12	Sigma-Aldrich (Saint Quentin Fallavier, France)	71-36-3
**Phenol>99% (PhOH)**	94.11	Sigma-Aldrich (Saint Quentin Fallavier, France)	108-95-2
**Chloroform D1 99,8% sans TMS ($CDCl_3$)**	120.38	Euriso top (Saint-Aubin, France)	965-49-6
**Methanol D4 99.8 (CD_3OD)**	36.07	Euriso top (Saint-Aubin, France)	811-98-3
**Dimethylsulfoxide D6 99.8% (DMSO-$_{d6}$)**	84.17	Euriso top (Saint-Aubin, France)	2206-27-1
**Acetonitrile for HPLC >99.8% (ACN)**	41.05	Fischer scientific (Waltham, Massachusetts, USA)	75-05-8
**Water from Mili-Q direct system; Resistivity 18.2 MΩ.cm (H_2O or water)**	18.02	Merck Millipore (Molsheim, France)	
**Phosphate buffer saline solution was obtained from dissolving one pellet in 200mL water yielding a solution of 0.01M phosphate buffer, 0.137M NaCl, 0.0027M KCl, pH 7.4 (PBS)**		Sigma-Aldrich (Saint Quentin Fallavier, France) (P4417)	
**Hyaluronic acid**	406,000	Givaudan (Pomacle, France)	
**Phosphatase, Alkaline from bovine intestinal mucosa**	160,000	Sigma-Aldrich (Saint Quentin Fallavier, France)	9001-78-9

2. Synthesis and characterization of Fmoc-FF*p*Y.

All peptides were prepared using solid support chemistry. The "Fmoc strategy" was used based on 2-CTC resin. The following synthetic pathway is given in the scheme below.

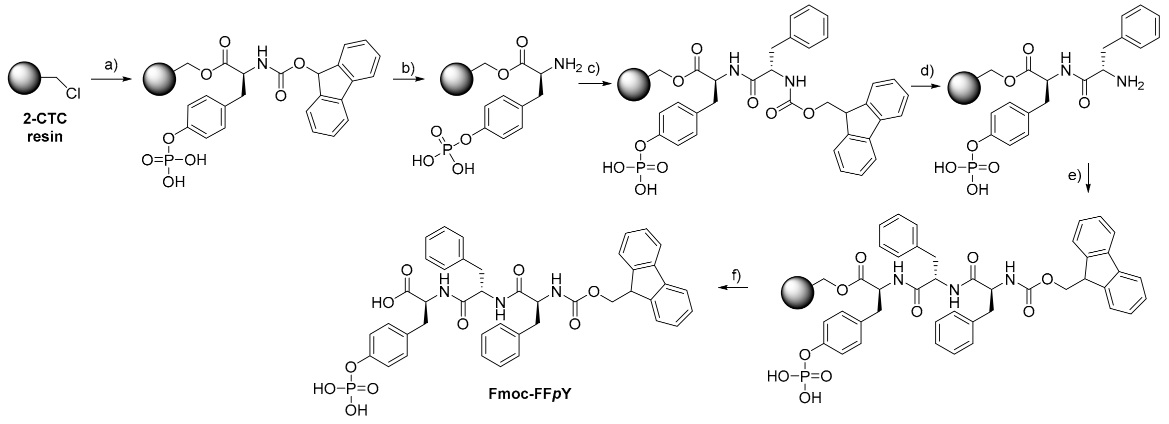

Scheme 1. Synthetic pathway to prepare Fmoc-FF*p*Y through solid support chemistry.

2.1. Experimental protocol.

a) 1,20g of 2-CTC resin (1.6mmol/g; 1.92 mmol) was stirred in a SPE tube in dry DCM (16mL) for 2h. DCM was filtered off and a solution of 2.76g Fmoc-$_{p}$Y-OH (5.76mmol) and DIEA (4mL) in DMF (16mL) was added. The tube was stirred overnight at room temperature and the solution was subsequently filtered off. Beads were washed twice with methanol and stirred for 1 hour at room temperature in 15mL of MeOH. The solution was then filtered off.

b) A solution of 20% piperidine in DMF (16mL) was added to the beads and the tube was stirred for 30 minutes. The solution was filtered off and the beads were washed 5 times with DCM (15mL). A Kaiser test* was performed on a small sample of beads and was positive (free amine present).

c) A solution of 2.23g Fmoc-F-OH (5.76mmol, 3eq), HBTU (2.19g, 3eq) and HOBt (790mg, 3eq) and DIEA (4mL) in DMF (14mL) was added. The tube was stirred overnight (15 hours) at room temperature. The solution was filtered off and the beads were washed 5 times with DCM (15mL). A Kaiser test* was performed on a small sample of beads and was negative (free amine absent).

d) A solution of 20% piperidine in DMF (12mL) was added to the beads and the tube was stirred for 20 minutes. The solution was filtered off and the beads were washed 5 times with DCM (15mL). A Kaiser test* was performed on a small sample of beads and was positive (free amine present).

e) A solution of 2.23g Fmoc-F-OH (5.76mmol, 3eq), HBTU (2.19g, 3eq) and HOBt (790mg, 3eq) and DIEA (4mL) in DMF (14mL) was added. The tube was stirred overnight (15 hours) at room temperature. The solution was filtered off and the beads were washed 5 times with DCM (15mL). A Kaiser test* was performed on a small sample of beads and was negative (free amine absent).

f) A solution of 95% TFA, 2.5% H_2O and 2.5% $HSi(iPr)_3$ (12mL) was added to the beads and the tube was stirred for 2 hours. The filtrate was recovered and evaporated until a thick oil is obtained. Et_2O (25mL) was added onto the oil and the mixture was cooled to 4°C for 5h. The resulting precipitate was recovered as a white powder (495mg ; 630 mmol ; 33% vs resin).

* Kaiser test protocol: (Ninhydrin Test): one drop of solution A, B and C is added in a test tube containing 10 beads of the resin. The test tube is heated at 100 °C.

Solution A:

- Dissolve 16.5 mg of KCN in 25 mL of distilled water.
- Dilute 1.0 mL of above solution with 49 mL of pyridine.
- Pour it into a small reagent bottle and label it "A"

Solution B:

- Dissolve 1.0 g of ninhydrin in 20 mL of BuOH.
- Pour into a small reagent bottle and label it as "B".

Solution C:

- Dissolve 40 g of PhOH in 20 mL of BuOH.
- Pour it into a small reagent bottle and label it "C".

When the Kaiser test is positive, the beads and the solution takes a blue coloration. This indicates the presence of free amine that means that the Fmoc deprotection step succeeds or a failed coupling step.

When the Kaiser test is negative, the beads and solution stays fairly uncolored. This indicates that the coupling step is completed or a failed Fmoc deprotection.

In case of a Fmoc deprotection or a coupling step failed, the step is repeated after a rinsing step until the Kaiser test yields the appropriate color.

2.2. Characterization of Fmoc-FFpY: ^{1}H, ^{13}C, ^{31}P and HPLC analyses.

2.2.1. H, ^{13}C and ^{31}P NMR of Fmoc-FFpY

NMR spectra were recorded on a Bruker Avance 400 spectrometer in the specified solvent at 25°C. The spectra were internally referenced to the residual solvent signal for ^{1}H and ^{13}C spectra and to the spectrometer internal reference for ^{31}P spectra. The chemical shifts were given in ppm and coupling constants J were listed in Hz. The following notation was used for the description of ^{1}H-NMR spectra: singlet (s), doublet (d), triplet (t), multiplet (m). Assignments were given as an indication from analysis of measured chemical shifts and signal multiplicity.

^{1}H NMR (400 MHz, CDCl3+10%MeOD) δ (ppm) = 2.84 (m, 2H), 2.97 (m, 2H), 3.02 (m, 2H), 4.09 (m, 1H), 4,17 (m, 1H), 4.31 (m, 2H), 4.50 (m, 1H), 4.57 (m, 1H), 7.02 (m, 2H), 7.04 (m, 2H), 7.09-7.18 (m, 10H), 7.23 (m, 2H), 7.34 (t, 2H, J=7 Hz), 7.44 (t, 2H, J=7 Hz), 7.70 (d, 2H, J=7 Hz).

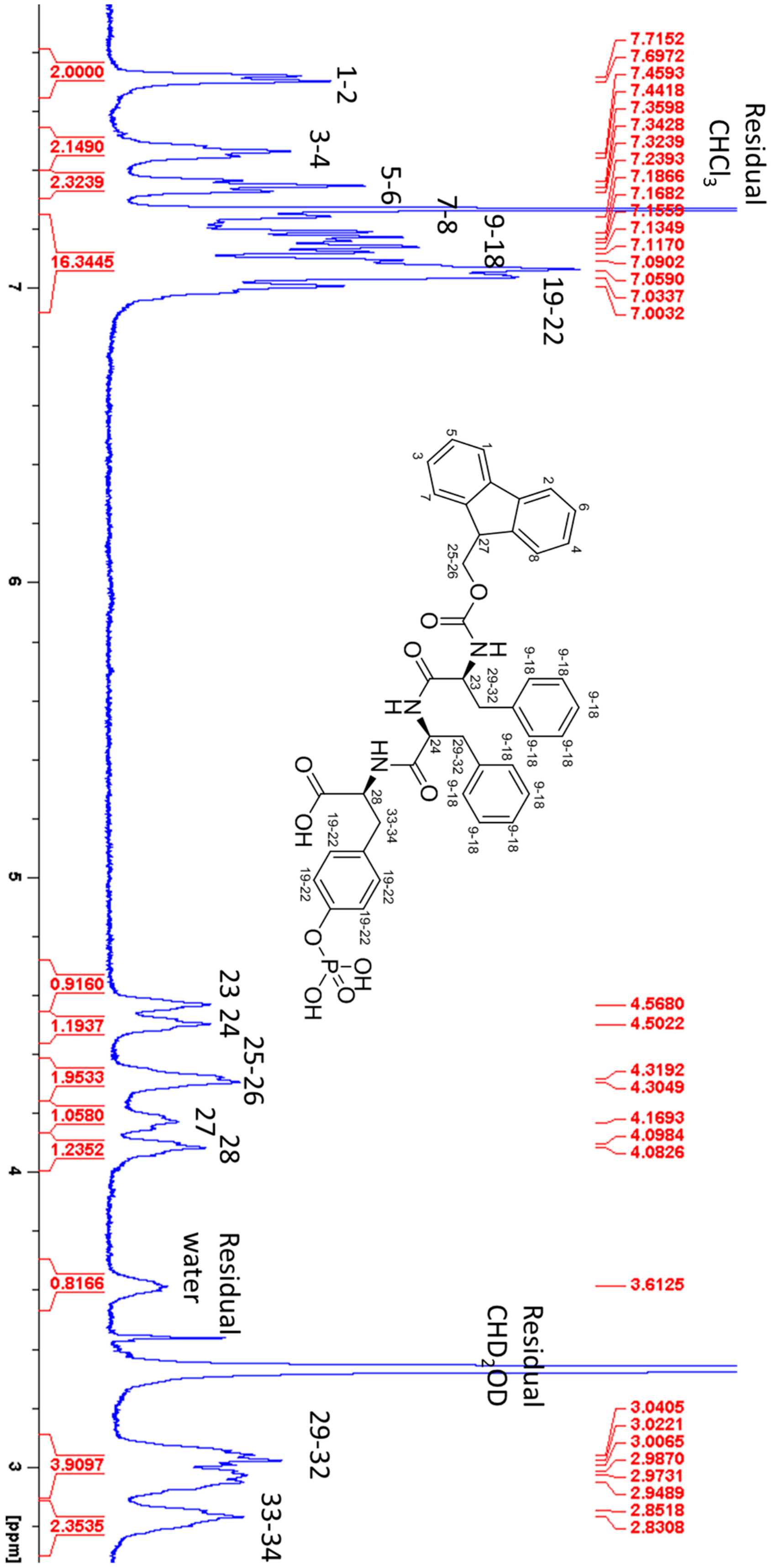

Figure S1. ^{1}H NMR spectra of Fmoc-FF*p*Y (400 MHz, $CDCl_3$+10%MeOD).

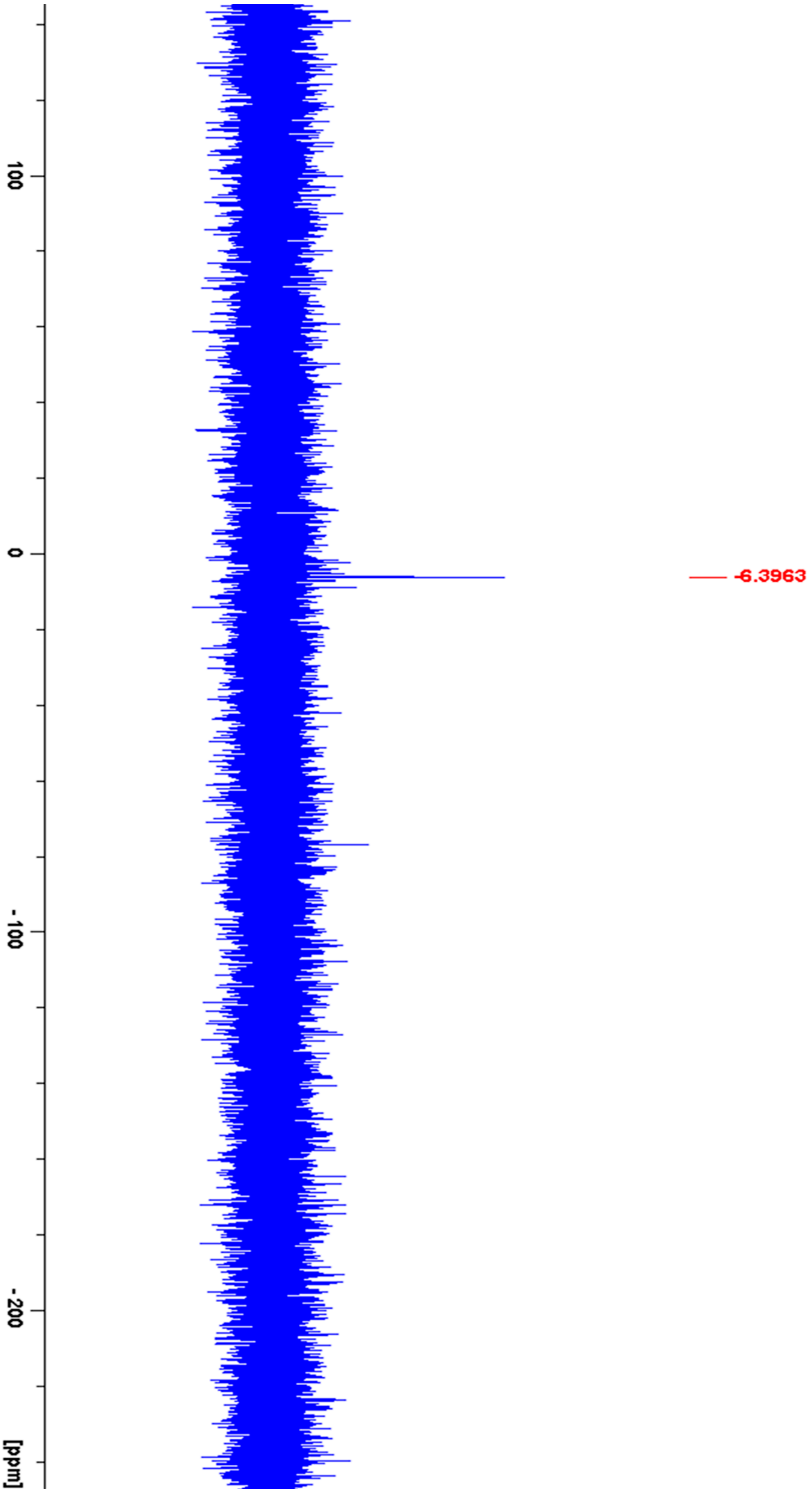

Figure S2. ^{31}P NMR spectra of Fmoc-FF*p*Y (161.92 MHz, $CDCl_3$+10% MeOD).

(a)

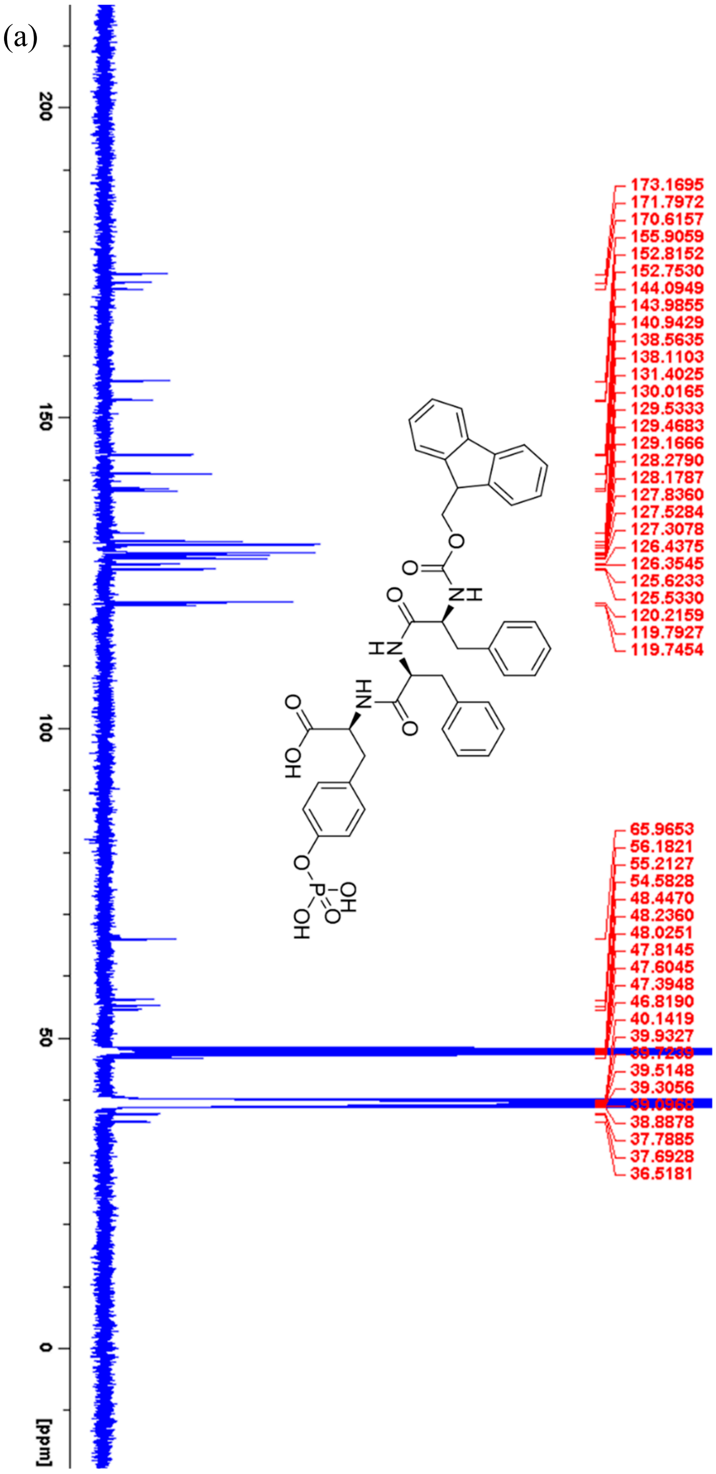

173.1695
171.7972
170.6157
155.9059
152.8152
152.7530
144.0949
143.9855
140.9429
138.5635
138.1103
131.4025
130.0165
129.5333
129.4683
129.1666
128.2790
128.1787
127.8360
127.5284
127.3078
126.4375
126.3545
125.6233
125.5330
120.2159
119.7927
119.7454
65.9653
56.1821
55.2127
54.5828
48.4470
48.2360
48.0251
47.8145
47.6045
47.3948
46.8190
40.1419
39.9327
39.5148
39.3056
38.8878
37.7885
37.6928
36.5181
200
150
100
50
0
[ppm]

(b)

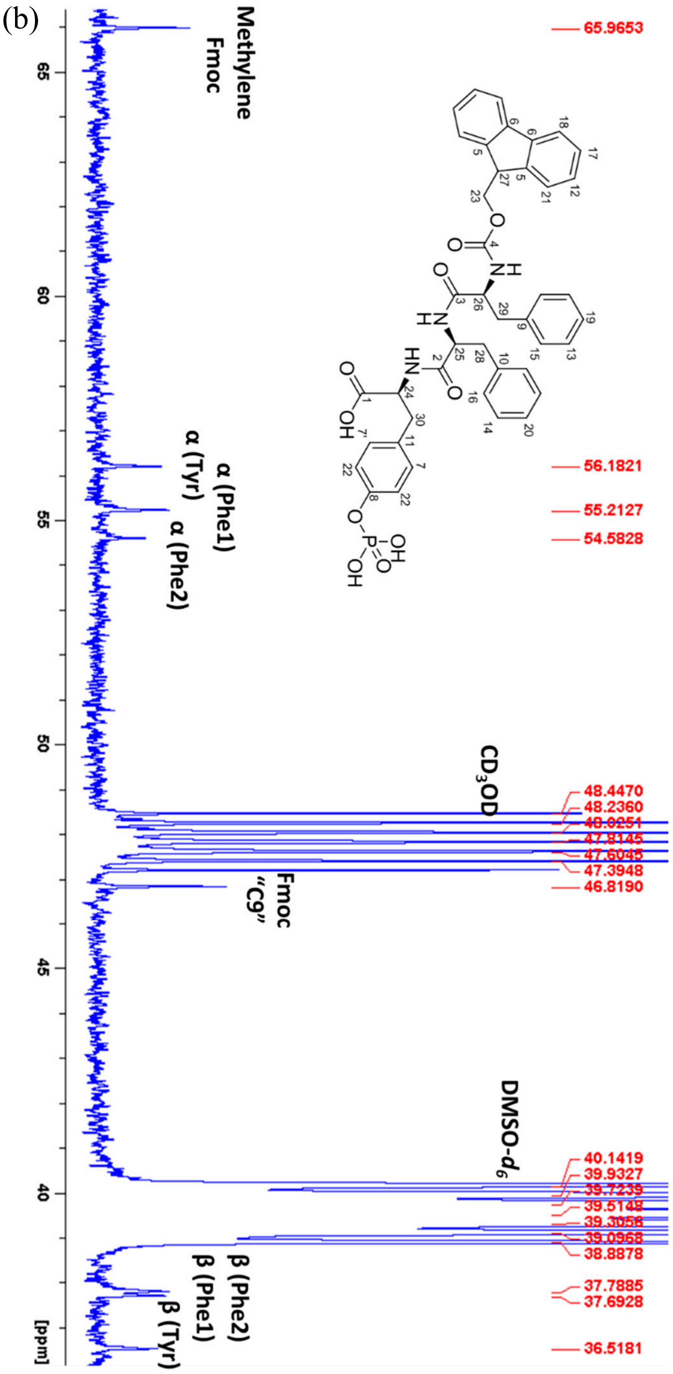

Methylene
Fmoc
α (Tyr)
α (Phe1)
α (Phe2)
CD3OD
Fmoc
"C9"
DMSO-d6
β (Phe2)
β (Phe1)
β (Tyr)
[ppm]
65.9653
56.1821
55.2127
54.5828
48.4470
48.2360
48.0251
47.8145
47.6045
47.3948
46.8190
40.1419
39.9327
39.7239
39.5148
39.3056
39.0968
38.8878
37.7885
37.6928
36.5181

(c)

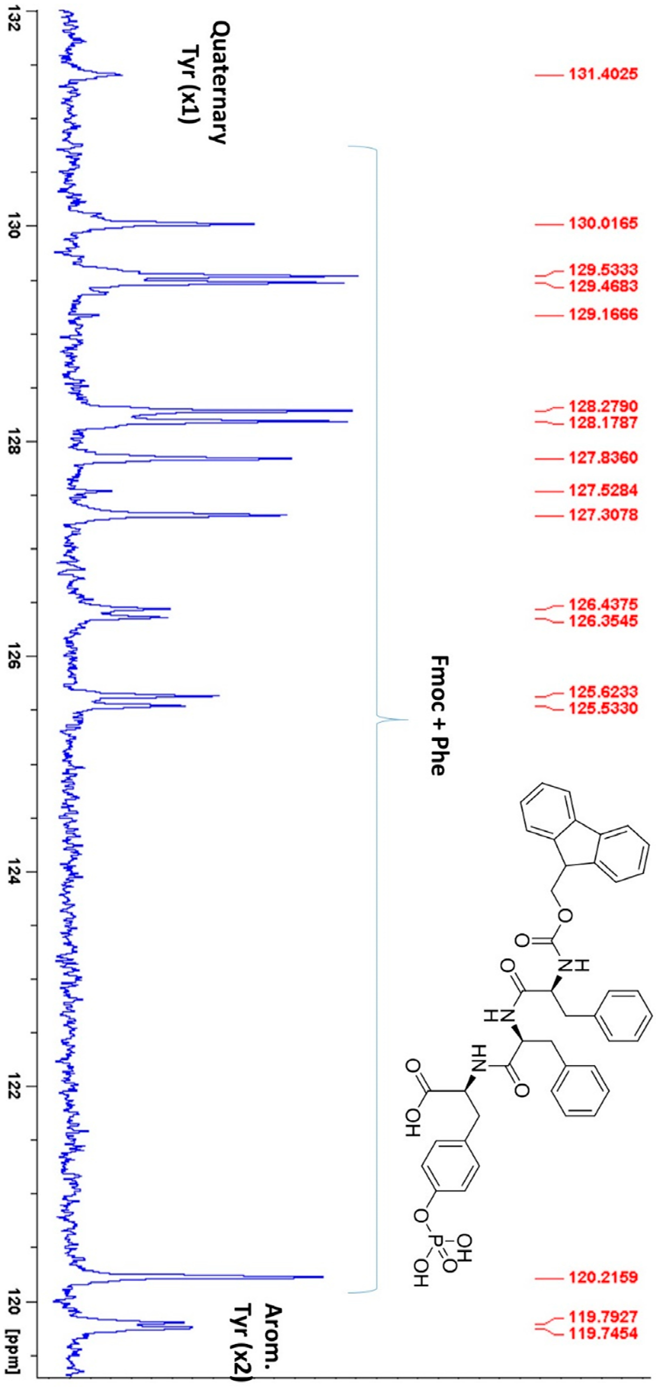

Quaternary Tyr (x1)
Fmoc + Phe
Arom. Tyr (x2)
131.4025
130.0165
129.5333
129.4683
129.1666
128.2790
128.1787
127.8360
127.5284
127.3078
126.4375
126.3545
125.6233
125.5330
120.2159
119.7927
119.7454
132
130
128
126
124
122
120 [ppm]

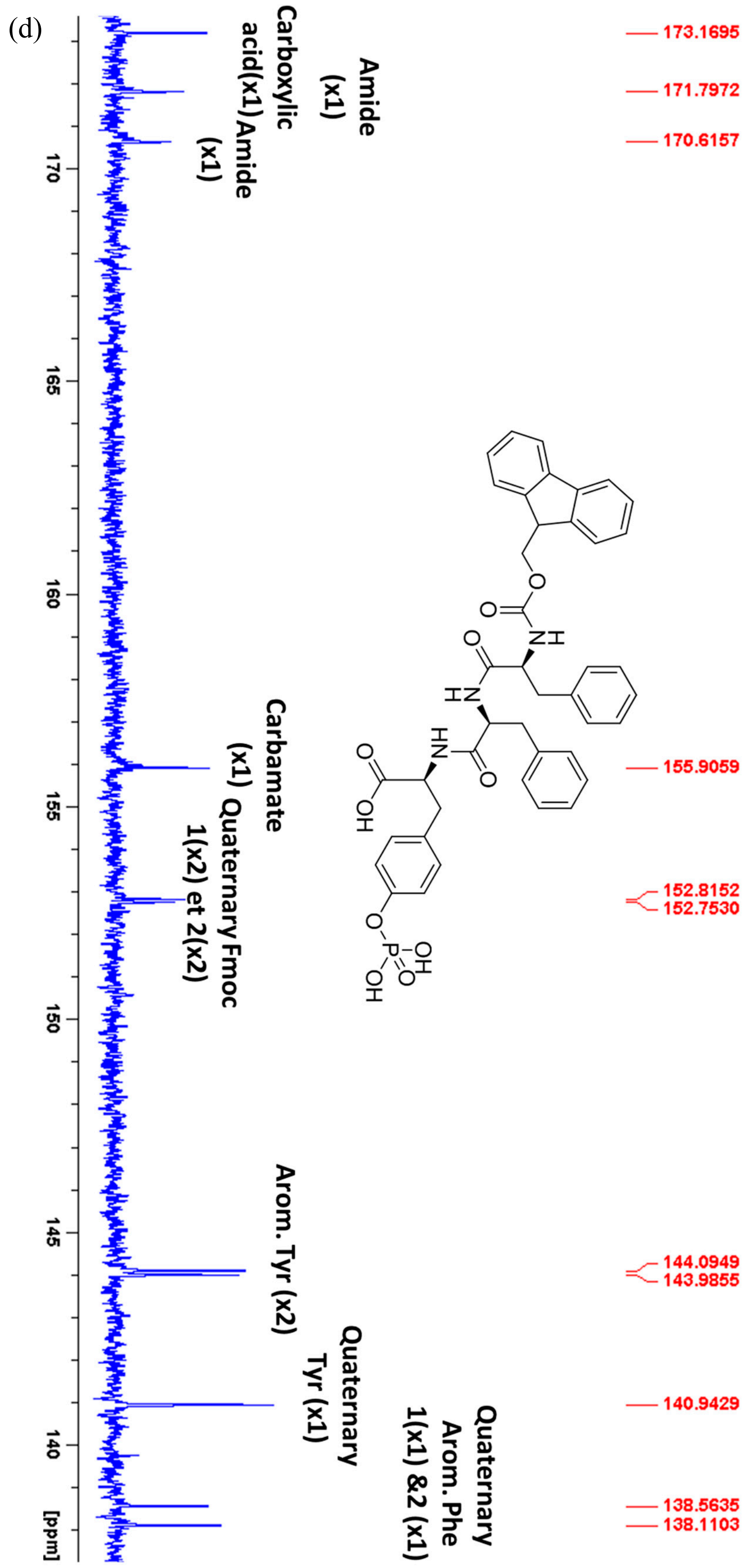

Figure S3. (**a**) ^{13}C NMR spectra of Fmoc-FF*p*Y (100 MHz, DMSO-d_6+10% MeOD) and magnifications. ^{13}C NMR (100 MHz, DMSO-d_6+10% MeOD) (**b**) δ(ppm) = 36.52, 37.69, 37.79, 46.82, 54.58, 55.21, 56.18, 65.97; (**c**) 119.74, 119.79, 120.22, 125.53, 125.62, 126.35, 126.44, 127.31, 127.53, 127.84,128.18, 128.28, 129.17, 129.47, 129,53, 130.02, 131.40; (**d**) 138.11, 138.56, 140.94, 143.99, 144.09, 152.75, 152.82, 155.91, 170.62, 171.80, 173.16 ppm.

2.2.2. HPLC of Fmoc-FFpY

HPLC experiments were carried out with a 1100 Series from Agilent technologies. Chromatograms were recorded and analysed by the software OpenLab Agilent 1100. Solutions were prepared in 10 mM PBS aqueous buffer (pH = 7.4) and all solutions are filtrated with a RC 0,2 µm filter before each injection.

Stationary phase: Interchim Uptsisphere 5µm C18HP; size 100 × 4.6mm.

Elution conditions: isocratic ACN/H_2O+0.1%TFA (60/40); Flow rate: 1 mL/min.

Temperature: 25 °C

Injection: 10 µL

Sample concentration: 0.5 mg/mL

Measured retention time: 3.03 min (=2.83× column dead volume)

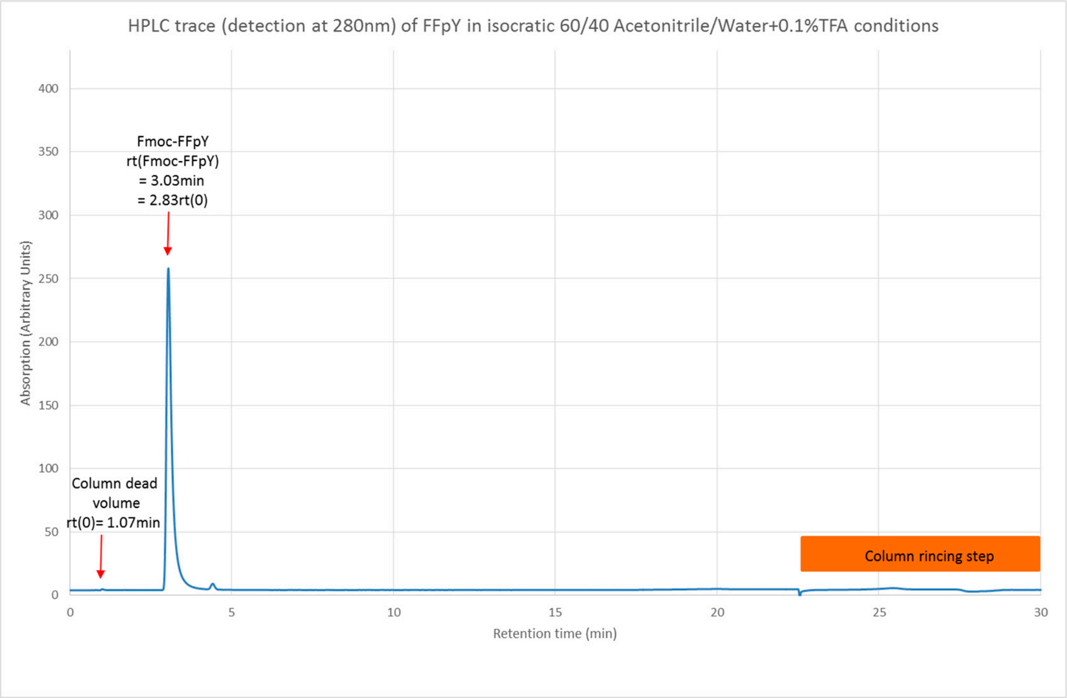

Figure S4. Chromatogram of the purified Fmoc-FF*p*Y.

3. Characterization of Hyaluronic acid (HA)

3.1. Size-exclusion Chromatography (SEC)

Size-exclusion Chromatography of hyaluronic acid was carried out on the DIONEX HPLC Ultimate 3000 system (degasser, pump, autosampler). The stationary phase were 4 Shodex OH-pak column of 30 cm (802.5HQ, 804HQ, 806HQ, 807HQ) with a guard column. The overall separation range is 300 to 100,000,000 g/mol. The eluant phase was water (Millipore quality) with 0.1 M $NaNO_3$ with a flow rate of 0.5 mL/min. The detector is a light scattering detector DAWN HELEOS II from Wyatt Techn with a dn/dc of 0.145 mL/g for hyaluronic acid. The hyaluronic acid was diluted in the eluant under agitation for 24h with 1h of heating at 50°C in order to remove possible aggregates. Then, the sample was filtrated on Millex filter of 0.45 µm and 50 µL was injected in the column for analysis. **The following values has been determined: Mn = 258,000 g/mol and Mw = 406,000 g/mol (polydispersity 1.574).**

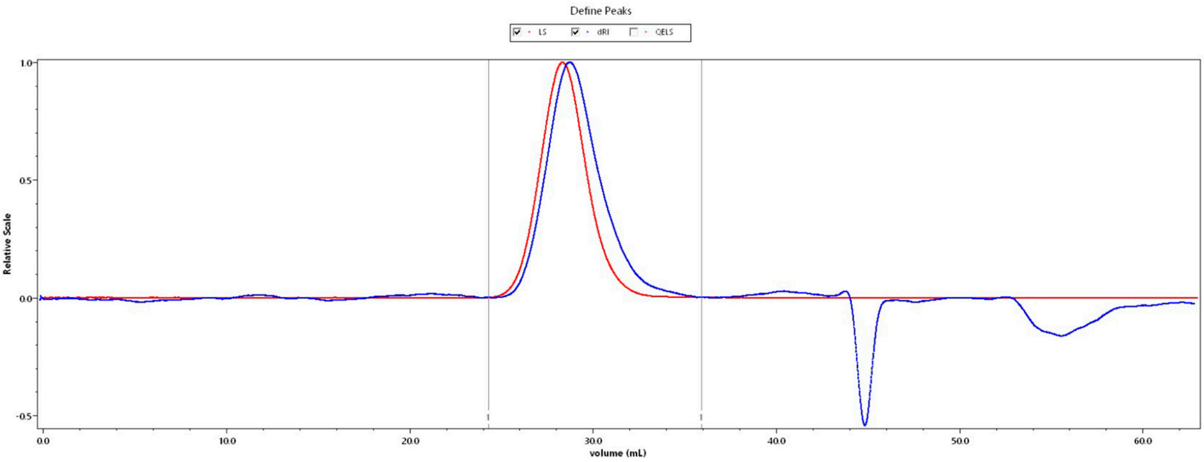

Figure S5. Size exclusion chromatogram of HA. The detection was done by light scattering (red line) and refractive index (blue line) measurements.

3.2.1. H NMR spectra of HA (D_20, 400 MHz)

^{1}H NMR spectra were recorded on a Bruker Avance 400 spectrometer in D_2O at 25°C. The spectra were internally referenced to the residual solvent signal for ^{1}H spectra (HDO at 4.75 ppm).

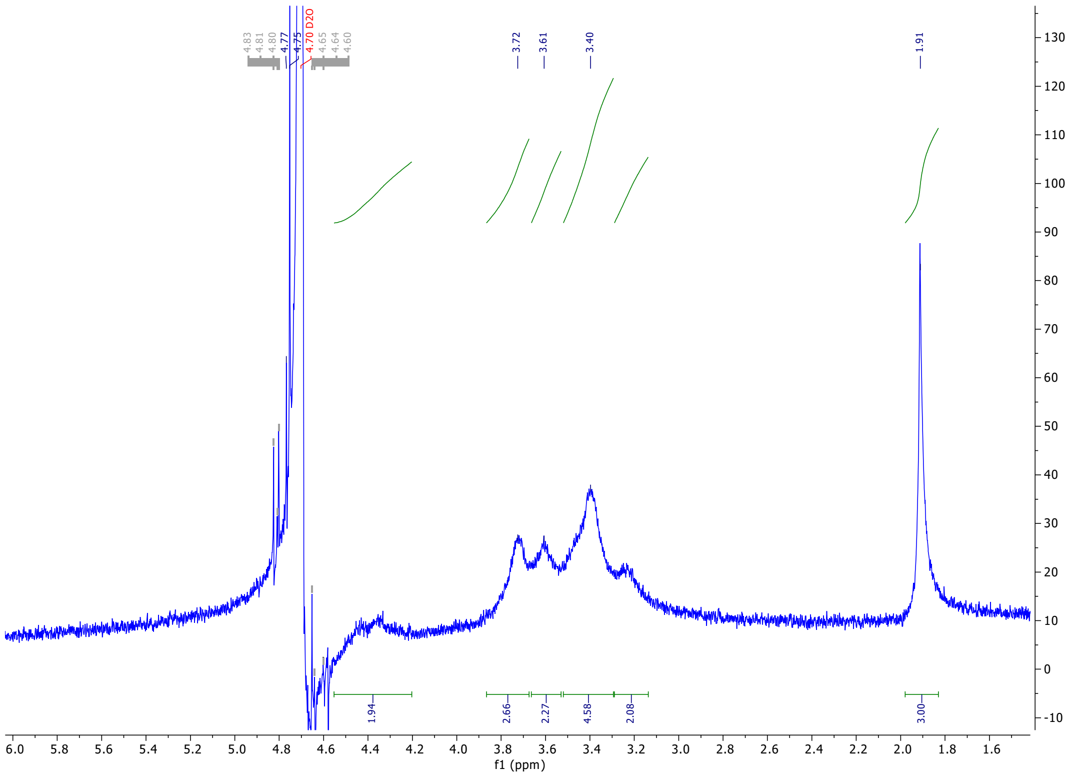

Figure S6. ^{1}H NMR (400 MHz) spectra of HA in D_2O at 25 °C.

4. Influence of HA on mechanical properties of Fmoc-FFY hydrogels by rheology

Elastic modulus (G′) and loss modulus (G′′) of Fmoc-FFY hydrogels obtained by dephosphoryation of the precursor peptide Fmoc-FFpY in presence of AP was evaluated in absence or in presence of HA by oscillatory dynamic rheology. Firstly, strain sweeps were performed to determine the linear viscoelastic regime (Figure a). Then, frequency sweeps were carried out at 0.06% strain (Figure b) and results show that G′ is higher than G′′ for all samples under study meaning the hydrogels are formed in all conditions. A decrease of the elastic modulus is exhibited when the HA concentration is increased.

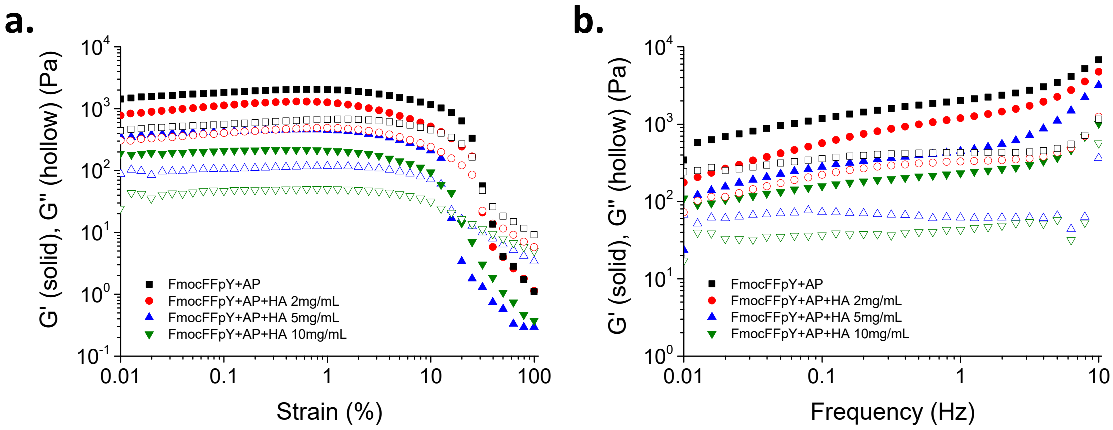

Figure S7. Evolution of the elastic modulus (G′) and loss modulus (G′′) as a function of the strain (**a**) or the frequency (**b**) for Fmoc-FF*p*Y and AP hydrogels in absence or in presence of HA.

5. Influence of HA on the peptide Fmoc-FFY self-assembly studied by CD

Circular dichroism (CD) spectra measurements were performed on a Jasco CD spectrometer (Model J-1700) using a quartz-cell of 1 cm path length. The following parameters were used for all experiments: scan speed of 20 nm/mn, resolution of 200 mdeg, Data Integration Time of 2 seconds, data pitch at 0,05 nm and band width at 2 nm. The baseline was automatically carried out with the 25 mM Borax buffer solution.

All solutions were prepared in a fresh 25 mM Borax buffer solution at pH 9.5: Fmoc-FF*p*Y solutions (0,75 mg/mL and 0,50 mg/mL), AP solutions (3x10^{-2} mg/mL and 2x10^{-2} mg/mL) and a HA solution (0,75 mg/mL).

Solutions were introduced in a specific order (HA, Fmoc-FF*p*Y and then AP) before being placed into the Circular Dichroism spectrometer.

The experiment was realized with 400 µL of the 0,75 mg/mL HA solution, 400 µL of the 0,75 mg/mL Fmoc-FF*p*Y solution and 400 µL of the 3x10^{-2} mg/mL AP solution, directly added in the quartz cell. Then it is quickly mixed by shaking the cell, and finally put into the spectrometer. The final concentrations into the cell are: 0,25 mg/mL for the Fmoc-FF*p*Y and 1×10^{-2} mg/mL for the AP.

The second experiment was carried out with 700 µL of the 0,50 mg/mL FmocFF*p*Y solution and 700 µL of the 2×10^{-2} mg/mL AP solution, directly added in the quartz cell. Then it is quickly homogenized and finally put into the cell holder. The final concentrations into the cell are: 0,50 mg/mL for the HA, 0,25 mg/mL for the Fmoc-FF*p*Y and 1×10^{-2} mg/mL for the AP.

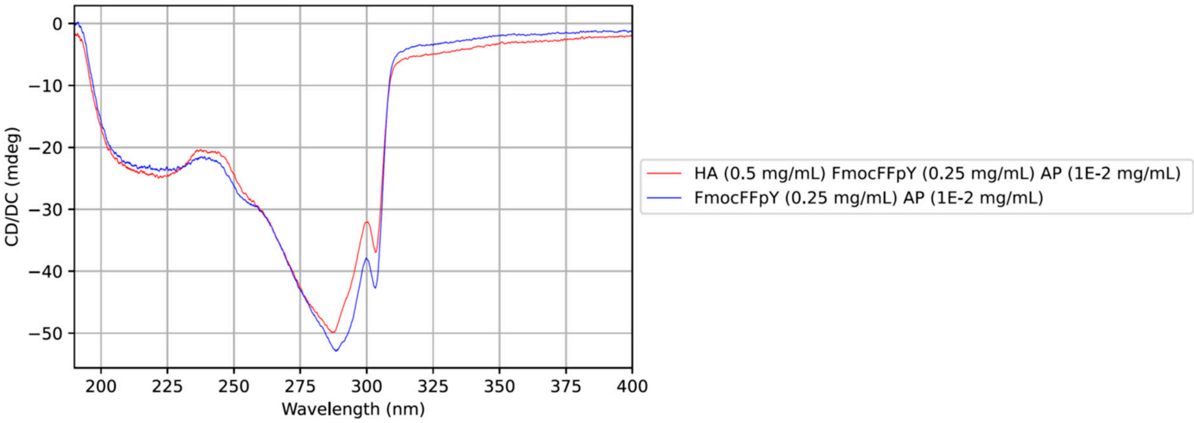

Figure S8. CD spectra of Fmoc-FF*p*Y and AP in absence (blue line) or in presence (red line) of HA.

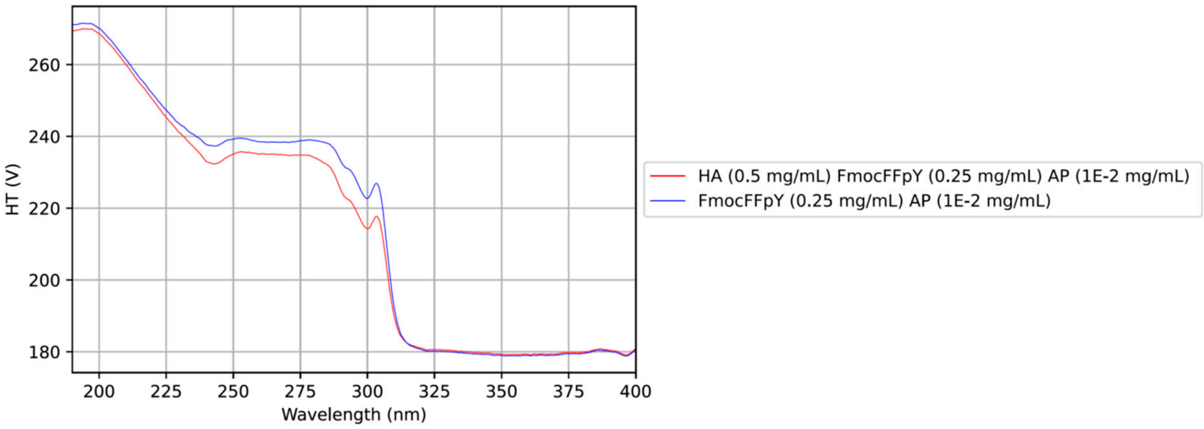

Figure S9. HT evolution corresponding to the CD spectra of Fmoc-FF*p*Y and AP in absence (blue line) or in presence (red line) of HA.
